# Clinical presentations and molecular basis of complement C1R mutation in a large turkish family

**DOI:** 10.1186/1546-0096-12-S1-P116

**Published:** 2014-09-17

**Authors:** Erkan Demirkaya, Ozgur Kasapcopur, Kenan Barut, Salim Caliskan, Adem Polat, Yesim Onen, Ivona Aksentijevich

**Affiliations:** 1 Inflammatory Disease Section, National Human Genome Research Institute, Bethesda, USA; 2 Pedaitric Rheumatology, İstanbul University, Cerrahpasa Medical Faculty, İstanbul Turkey, Istanbul, USA; 3 Pedaitric Rheumatology, İstanbul University, Cerrahpasa Medical Faculty, İstanbul Turkey, Istanbul, Turkey; 4 FMF Arthritis Vasculitis and Orphan disease Research in pediatric rheumatology (FAVOR), Gulhane Military Medical Academy, Ankara, Turkey

## Introduction

Inherited deficiencies of complement components can result in autoimmunity. Early age of onset, prominent cutaneous manifestations, and presence of anti-Ro antibodies are features suggestive of a complement deficiency. SLE-associated deficiencies in subcomponents of the C1 complex, C1r and/or C1s, were described 4 decades ago, however the molecular basis and functional aspects of the complement deficiency have not been clearly determined.

## Objectives

The aim of this study was to identify the cause of presumably recessively inherited form of SLE and/or lupus-like syndrome (LLS) in a consanguineous family from Turkey.

## Methods

We studied 2 pairs of young female siblings(4 patients), who presented with Lupus-like syndrome but with a significant phenotypic variability and differences in the disease severity. They shared a history of episodes of malar and/or generalized rash and ANA positivity, while anti-dsDNA were negative in all four. Renal involvement was prominent in one pair of siblings and one patient developed CNS manifestations including convulsion.

DNA samples from the affected patients, their unaffected parents and siblings were isolated from whole blood. We performed whole-exome sequencing in 9 samples from this family and Sanger sequencing in other family members.

## Results

We identified a homozygous frameshift mutation in the *CR1* gene (NM_001733.4;c.1331delT; p.Pro445Leufs*11), encoding complement 1r subunit, in all 4 affected siblings. The homozygous p.Pro445Leufs*11 mutation was validated with Sanger sequencing in all four patients while their unaffected parents and siblings were either heterozygous carriers or non-carriers. One 9y old sibling was identified as homozygous for the mutation but is yet unaffected. Despite the same genotype the affected patients have variable phenotypes concerning disease features, severity, disease outcome, thus suggesting a role for other modifying alleles or epigenetic factors.

## Conclusion

Our findings show the second molecular evidence that loss-of-function mutations in C1R are the cause of SLE or lupus-like syndrome. We report a novel genetic defect in the C1r complement protein leading to a recessive form of familial SLE/LLS in the Turkish population. This mutation is likely present in the general population and it should be included in diagnostic evaluation of Turkish patients with early onset SLE.

We plan extensive biochemical, immunologic, and functional assays to assess the impact of this pathogenic mutation on complement function, apoptosis, neutrophil and B cell biology.

## Disclosure of interest

None declared.

